# Identification of Non-HLA Genes Associated with Celiac Disease and Country-Specific Differences in a Large, International Pediatric Cohort

**DOI:** 10.1371/journal.pone.0152476

**Published:** 2016-03-25

**Authors:** Ashok Sharma, Xiang Liu, David Hadley, William Hagopian, Edwin Liu, Wei-Min Chen, Suna Onengut-Gumuscu, Ville Simell, Marian Rewers, Anette-G. Ziegler, Åke Lernmark, Olli Simell, Jorma Toppari, Jeffrey P. Krischer, Beena Akolkar, Stephen S. Rich, Daniel Agardh, Jin-Xiong She, TEDDY Study Group

**Affiliations:** 1 Center for Biotechnology and Genomic Medicine, Georgia Regents University, Augusta, GA, United States of America; 2 Pediatric Epidemiology Center, Department of Pediatrics, University of South Florida, Tampa, FL, United States of America; 3 Division of Population Health Sciences and Education, St George's University of London, London, United Kingdom; 4 Pacific Northwest Diabetes Research Institute, Seattle, WA, United States of America; 5 Digestive Health Institute, Children’s Hospital Colorado, University of Colorado Denver, Aurora, CO, United States of America; 6 Center for Public Health Genomics, University of Virginia, Charlottesville, VA, United States of America; 7 Department of Pediatrics, University of Turku, Turku, Finland; 8 Barbara Davis Center for Childhood Diabetes, University of Colorado Denver, Aurora, CO, United States of America; 9 Institute of Diabetes Research, Helmholtz Zentrum München, and Klinikum rechts der Isar, Technische Universität München, and Forschergruppe Diabetes e.V., Munich-Neuherberg, Germany; 10 Department of Clinical Sciences, Lund University/CRC, Malmö, Sweden; 11 National Institutes of Diabetes and Digestive and Kidney Disorders, National Institutes of Health, Bethesda, MD, United States of America; 12 Diabetes and Celiac Disease Unit, Lund University, Malmo, Sweden; National Taiwan University, TAIWAN

## Abstract

**Objectives:**

There are significant geographical differences in the prevalence and incidence of celiac disease that cannot be explained by HLA alone. More than 40 loci outside of the HLA region have been associated with celiac disease. We investigated the roles of these non-HLA genes in the development of tissue transglutaminase autoantibodies (tTGA) and celiac disease in a large international prospective cohort study.

**Methods:**

A total of 424,788 newborns from the US and European general populations and first-degree relatives with type 1 diabetes were screened for specific HLA genotypes. Of these, 21,589 carried 1 of the 9 HLA genotypes associated with increased risk for type 1 diabetes and celiac disease; we followed 8676 of the children in a 15 y prospective follow-up study. Genotype analyses were performed on 6010 children using the Illumina ImmunoChip. Levels of tTGA were measured in serum samples using radio-ligand binding assays; diagnoses of celiac disease were made based on persistent detection of tTGA and biopsy analysis. Data were analyzed using Cox proportional hazards analyses.

**Results:**

We found 54 single-nucleotide polymorphisms (SNPs) in 5 genes associated with celiac disease (*TAGAP*, *IL18R1*, *RGS21*, *PLEK*, and *CCR9*) in time to celiac disease analyses (10^−4^>*P*>5.8x10^−6^). The hazard ratios (HR) for the SNPs with the smallest P values in each region were 1.59, 1.45, 2.23, 2.64, and 1.40, respectively. Outside of regions previously associated with celiac disease, we identified 10 SNPs in 8 regions that could also be associated with the disease (*P*<10^−4^). A SNP near *PKIA* (rs117128341, *P* = 6.5x10^−8^, HR = 2.8) and a SNP near *PFKFB3* (rs117139146, *P*<2.8x10^−7^, HR = 4.9) reached the genome-wide association threshold in subjects from Sweden. Analyses of time to detection of tTGA identified 29 SNPs in 2 regions previously associated with celiac disease (*CTLA4*, *P* = 1.3x10^−6^, HR = 0.76 and *LPP*, *P* = 2.8x10^−5^, HR = .80) and 6 SNPs in 5 regions not previously associated with celiac disease (*P*<10^−4^); non-HLA genes are therefore involved in development of tTGA.

**Conclusions:**

In conclusion, using a genetic analysis of a large international cohort of children, we associated celiac disease development with 5 non-HLA regions previously associated with the disease and 8 regions not previously associated with celiac disease. We identified 5 regions associated with development of tTGA. Two loci associated with celiac disease progression reached a genome-wide association threshold in subjects from Sweden.

## Introduction

Celiac disease is strongly associated with the human leukocyte antigen (HLA) DR3–DQ2.5 (*i*.*e*., DRB1*03-DQA1*05:01-DQB1*02:01) or DR4-DQ8 (DRB1*04-DQA1*03-DQB1*03:02) haplotypes on chromosome 6 [1]. Moreover, there is an HLA gene-dose effect on the disease risk as individuals carrying two copies of DR3-DQ2.5 are at a higher susceptibility for celiac disease than those with only one copy [2,3]. Although carrying either DR3–DQ2.5 or DR4–DQ8 is almost a necessity to develop celiac disease, these haplotypes are common in the general population and not all carriers develop clinical disease [4]. Since the first genome-wide case/control association study (GWAS) on celiac disease was published in 2007, a total of 40 non-HLA loci have been suggested as being associated with celiac disease[5–9]. A significant proportion of the genetic predisposition comes from the HLA region (odds ratio of >5) while non-HLA genes have modest effect sizes with an odds ratio between 1.12 and 1.36 for celiac disease [10]. The role of these non-HLA genes have not been assessed in those with early onset celiac disease, particularly using a prospective cohort.

Celiac disease is increasing in frequency, with significant intra- and inter-country differences in the prevalence and incidence of the disease[11]. Despite recent advances in celiac disease genetics, it remains elusive why some, but not all, individuals with the HLA risk genotypes develop celiac disease. Although the ingestion of gluten is required to trigger and maintain celiac disease, gluten exposure is nearly universal. Therefore, exposures to other environmental factors may also be important in the pathogenesis. Celiac disease is likely a multifactorial disorder where multiple genes and multiple environmental factors interact in a complex manner. Disease risk genes may act at various stages of autoimmunity progression, with some genes playing a role early in autoantibody development, and others playing a critical role in the later stages of celiac disease development. This stage-specific contribution of different genes to the celiac disease risk is an important concept, which cannot be investigated using the cross-sectional case/control study design employed in all previous studies. Furthermore, genetic factors responsible for the development of tissue transglutaminase autoantibodies (tTGA) and ethnic- or country-specific differences in a genetically predisposed population have not been reported previously.

The Environmental Determinants of Diabetes in the Young (TEDDY) is an international multicenter study that screened over 420,000 newborns from the general population in four different countries to identify children with high risk HLA genes for the development of type 1 diabetes (T1D) [12]. Recently, TEDDY demonstrated the impact of different HLA genotypes on the risk of celiac disease as well as tTGA development, and furthermore confirmed that the HLA-DR3-DQ2/DR3-DQ2 genotype confers the single highest genetic risk for the disease during early childhood[13]. We also found differences in risk of disease between the participating countries that could not be explained by HLA-DR-DQ, suggesting that the risk may be influenced by variations in the environment and/or involvement of genes outside the HLA-DR-DQ region. One such recent finding from the TEDDY study was the protective association of HLA-DRB1*0401 with celiac disease autoimmunity[14].

The present study genotyped 195,806 SNPs on ImmunoChip in 6,010 TEDDY children to identify potential genetic factors responsible for the development of early autoimmunity (tTGA development) and celiac disease as well as country-specific differences in genetic predisposition.

## Results

A total of 703 subjects developed persistent tTGA and were considered “events” in the Cox proportional hazards models for persistent tTGA. Only 317 of these 703 persistently tTGA positive children received an intestinal biopsy and 262 of the 317 subjects were confirmed to have celiac disease at the time the procedure was performed. Eleven children with positive tTGA tests at the initial time point were biopsied before they could be confirmed as having persistent tTGA and eight of them also had biopsy-proven celiac disease. Eighteen other children who had persistent tTGA levels >100 units but did not have a biopsy were also considered to have celiac disease for purposes of the study. Therefore, 288 subjects were considered as “events” in the analysis of the time-to-celiac disease. There are known differences based upon the family history of celiac disease, HLA-DR-DQ genotype, gender, *HLA-DPB1* and country of residence. These factors were adjusted in the Cox proportional hazard models.

### Analysis of reported celiac disease SNPs

A total of 69 SNPs were previously reported to be associated with celiac disease based on the NHGRI GWAS Catalog[5,7,15–17], of which 48 were represented on the ImmunoChip (**S4 Table**). Risk Variants that have been reported but are not on the ImmunoChip are listed in **S6 Table**. In the time-to-celiac disease analysis, only one SNP (rs13015714/*IL18R* on 2q12.1, HR = 1.42, p = 1.38x10^-4^) attained significance after Bonferroni correction (p = 0.05/48 = 0.001). Several other SNPs were close to the significance threshold of 0.001 or had p-value <0.05: rs653178/SH2B3 (HR = 1.30; p = 0.002); rs1464510/*LPP* (HR = 1.28; p = 0.002); rs17035378/*PLEK* (HR = 0.75; p = 0.004); rs6806528/*FRMD4B* (HR = 1.44; p = 0.004); rs11221332/*ETS1* (HR = 1.29; p = 0.006); rs2298428/*YDJC* (HR = 1.27; p = 0.012); rs2327832/*TNFAIP3* (HR = 1.24; p = 0.025); rs802734/*PTPRK* (HR = 1.21; p = 0.034); rs13098911/CCR9 (HR = 1.29; p = 0.041); and rs10876993/CDK4 (HR = 0.84; p = 0.042).

For time-to-persistent tTGA analysis, we observed 10 SNPs with p<0.05: rs1464510/*LPP* (HR = 1.16; p = 0.004); rs2298428/*YDJC* (HR = 1.17; p = 0.011); rs864537/*CD247* (HR = 0.87; p = 0.013); rs13015714/*IL18R1* (HR = 1.15; p = 0.02); rs10936599/*MYNN* (HR = 1.15; p = 0.022); rs11203203/*UBASH3A* (HR = 1.13; p = 0.027); rs11712165/*CD80* (HR = 1.12; p = 0.035); rs7574865/*STAT4* (HR = 1.14; p = 0.036); rs2816316/*RGS1* (HR = 0.859; p = 0.038); and rs802734/*PTPRK* (HR = 1.12; p = 0.046).

### Analysis of previously reported celiac disease regions

**Fig 1:**
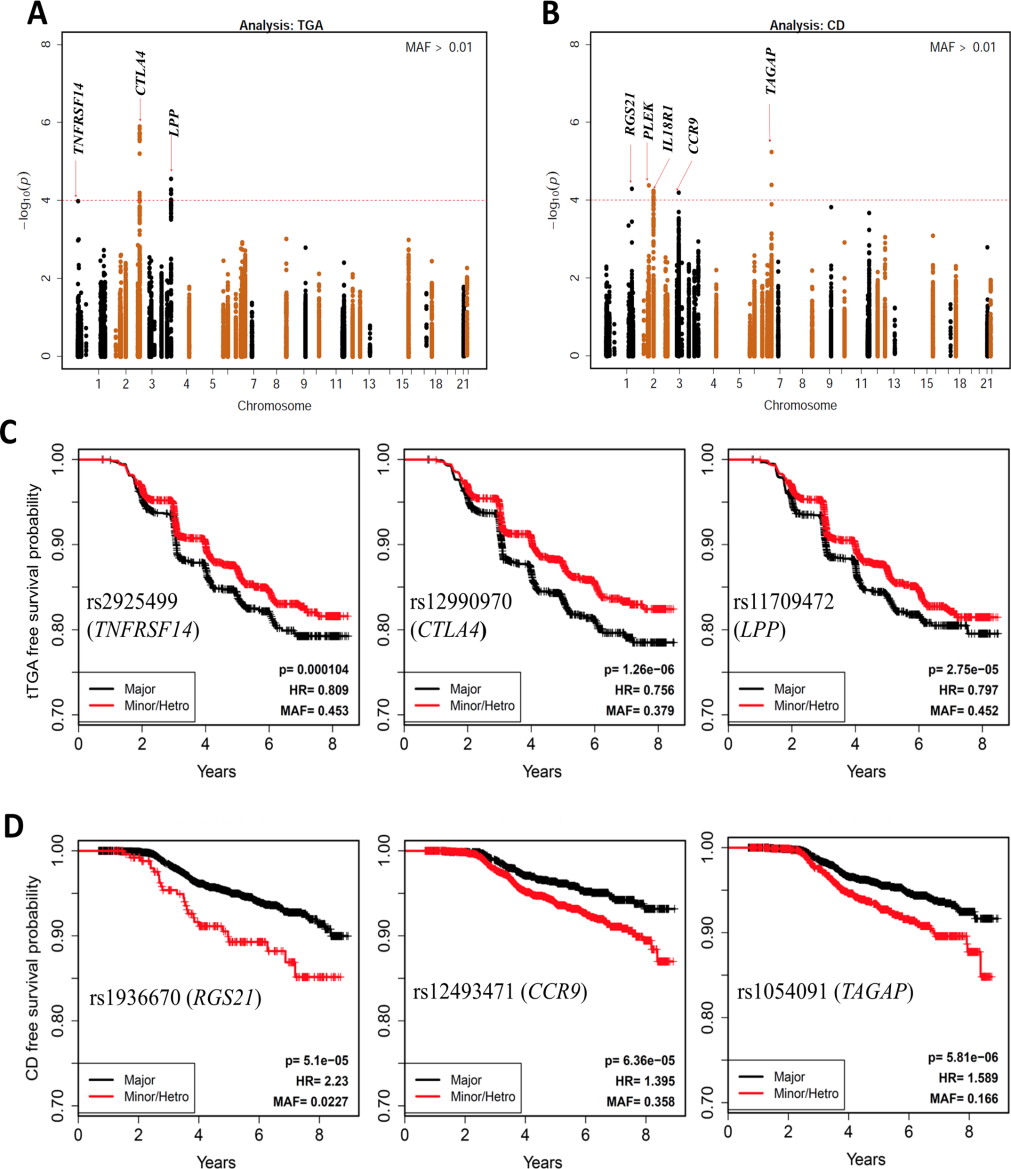
SNPs in the previously reported celiac disease associated regions. Manhattan plot of *P*-values on the −log_10_ scale for SNPs (±400kb) previously associated with celiac disease (**A**) and persistent tissue transglutaminase autoantibody (tTGA) positivity **(B**). HRs and p-values are calculated using three possible genotypes and adjusted for family history of celiac disease, HLA-DR-DQ genotype, gender, *HLA-DPB1*, population stratification (ancestral heterogeneity) and country of residence (as strata). The red dashed line represents *p* = 1x10^−4^. Kaplan-Meier plots of the three most significant SNPs associated with celiac disease (**C**) and tTGA (**D**) are plotted by dividing the subjects in two groups: (i) Major homozygous (black curves) and (ii) Heterozygous combined with minor homozygous (red curves).

Next, we extended our analysis to all SNPs within 400 kb up- and downstream of the 48 reported SNPs. The–log10 p-values for all SNPs in these regions are plotted in **Fig 1A** for tTGA and **Fig 1B** for celiac disease. Since these are analyses for candidate regions, we considered p<10^−4^ as suggestive evidence for confirmation because multiple SNPs are tested in each region and the SNPs are in high linkage disequilibrium.

In the tTGA plots, the two regions with strongest evidence were *CTLA4* and *LPP* (**S1 Fig**). The SNPs with smallest p-value in these two regions are: rs12990970/*CTLA4* (HR = 0.76; p = 1.3x10^-6^) and rs11709472/*LPP* (HR = 0.80; P = 2.8x10^-5^) (**Table 1**). It is important to note that in our study, the presence of the minor allele of rs12990970/*CTLA4* is protective (HR<1). In contrast, earlier studies have shown that *CTLA4* (rs4675374-A) is a risk factor for celiac disease (OR = 1.14)[5,18]. The Kaplan-Meier plots for the three SNPs with smallest p-values in time-to-tTGA analysis (rs12990970/*CTLA4*; rs11709472/*LPP;* rs2925499/*TNFRSF14*) are shown in **Fig 1C**.

**Table 1:** Associations with celiac disease or tissue transglutaminase autoantibody (tTGA) positivity (p<10^−4^), mapped to previously known regions.

SNP*^a^*	CHR	BP	MAF	HR CD	P-value*^b^* CD	HR tTGA	P-value^b^ tTGA	# SNPS (P<10^−4^)	Nearby Gene
rs1936670	1	190598185	0.02	2.23	**5.10x10**^**-5**^	1.36	0.038	1	*RGS21*
rs4851575	2	102391635	0.24	1.45	**5.69x10**^**-5**^	1.16	0.014	49	*IL18R1*, *IL18RAP*
rs114569351	2	68520426	0.02	2.64	**4.19x10**^**-5**^	1.73	0.002	1	*PLEK*,*FBXO48*
rs12493471	3	45926682	0.36	1.40	**6.36x10**^**-5**^	1.09	0.098	1	*CCR9*,*LZTFL1*,*CXCR6*
rs1054091	6	159389500	0.17	1.59	**5.81x10**^**-6**^	1.22	0.004	2	*RSPH3*,*TAGAP*
rs12990970	2	204408934	0.38	0.82	0.027	0.76	**1.26x10**^**-6**^	21	*NPM1P33*,*CTLA4*
rs11709472	3	189560280	0.45	0.82	0.019	0.80	**2.75x10**^**-5**^	8	*LPP*

CHR: Chromosome; BP: Base Pair Position (NCBI 36.3); MAF: Minor Allele Frequency; HRCD: Hazard Ratio in celiac disease analysis; HRtTGA: Hazard Ratio in tTGA analysis. P-values < 10^−4^ are highlighted in bold

^a^The data for the SNP with smallest p-value is presented from each region.

^b^HRs and p-value adjusted for family history of celiac disease, HLA-DR-DQ genotype, gender, *HLA-DPB1*, population stratification (ancestral heterogeneity) and country of residence (as strata).

In the celiac disease analysis, we found 5 regions (*TAGAP*, *IL18R1*, *RGS21*, *PLEK*, and *CCR9*) with SNPs that had p-values <10^−4^: rs1054091/*TAGAP* (HR = 1.59; p = 5.8x10^-6^); rs4851575/*IL18R1* (HR = 1.45; p = 5.7x10^-5^); rs1936670/*RGS21* (HR = 2.23; p = 5.1x10^-5^); rs114569351/PLEK (HR = 2.64; p = 4.2x10^-5^); and rs12493471/CCR9 (HR = 1.40; p = 6.4x10^-5^) (**Table 1, S2 Fig**). The Kaplan-Meier plots of three SNPs with smallest p-values in the time-to-celiac disease analysis (rs1936670/*RGS21*, rs12493471/*CCR9*, rs1054091/*TAGAP*) are shown in **Fig 1D**.

### SNPs associated with progression to persistent tTGA outside of the known celiac disease regions

**Fig 2:**
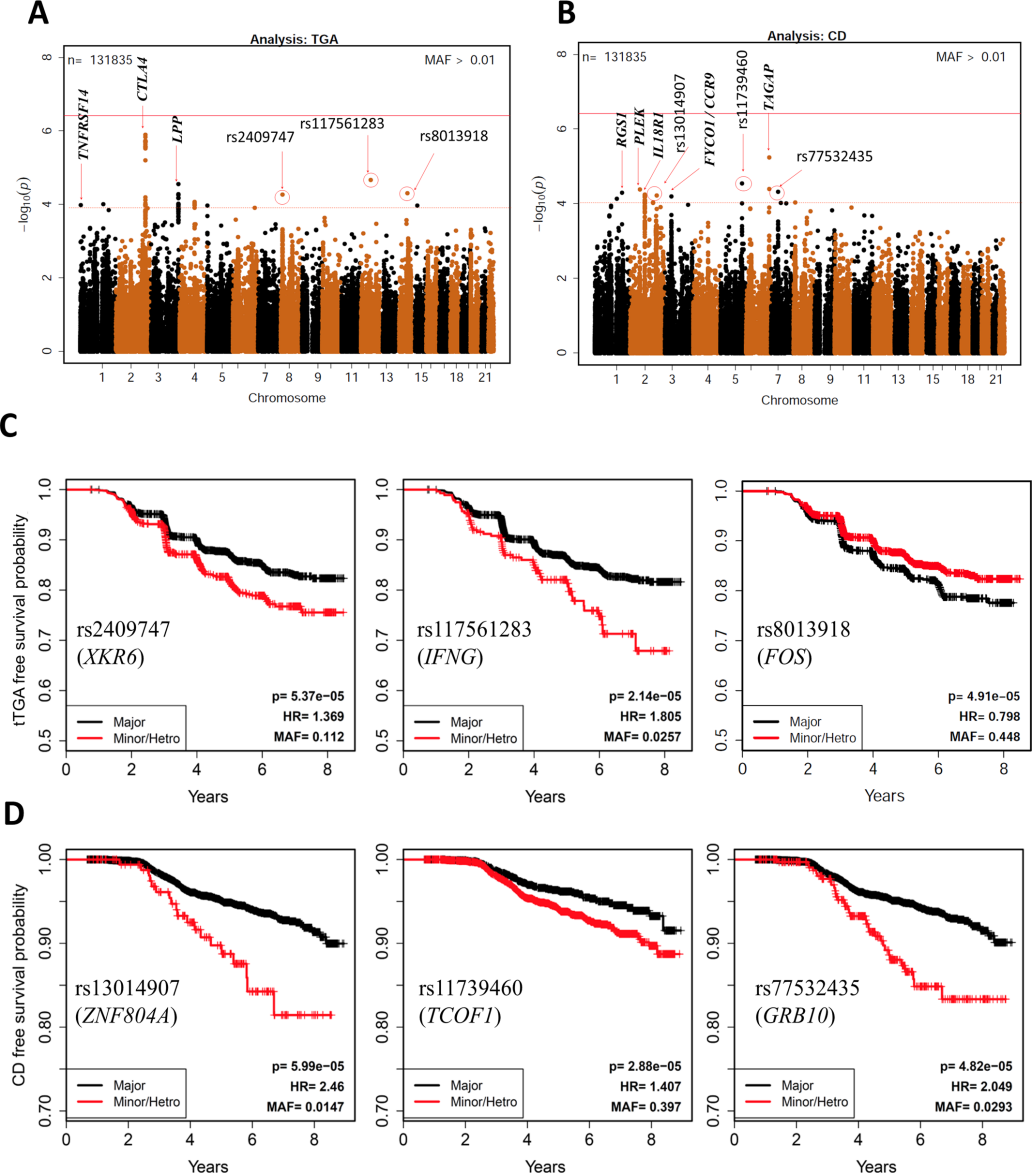
Associations with risk of celiac disease and risk of persistent tissue transglutaminase autoantibody (tTGA) positivity. Manhattan plot of 133,620 SNPs with MAF>0.01, displaying the *P*-values on the −log_10_ scale for SNP associations with celiac disease (**A**) and persistent tTGA positivity **(B**). HRs and p-values are calculated using three possible genotypes and adjusted for family history of celiac disease, HLA-DR-DQ genotype, gender, *HLA-DPB1*, population stratification (ancestral heterogeneity) and country of residence (as strata). The red dashed line represents *p* = 1x10^−4^, the red solid line represents Bonferroni correction threshold. Kaplan-Meier plots of selected SNPs associated with celiac disease (**C**) and persistent tTGA (**D**) are plotted by dividing the subjects in two groups: (i) Major homozygous (black curves) and (ii) Heterozygous combined with minor homozygous (red curves).

We then extended the analysis to include all SNPs genotyped on the ImmunoChip in search of novel SNP associations. For these analyses, 133,620 with minor allele frequencies of at least 0.01 were tested and therefore the statistical significance for any single SNP requires a Bonferroni-corrected p<3.7x10^-7^. In the time-to-persistent tTGA analyses, none of the SNPs reached this significance threshold, but 7 SNPs were identified in 5 novel celiac disease regions with p<10^−4^: rs117561283/*IFNG* (HR = 1.81; p = 2.1x10^-5^); rs8013918/*FOS* (HR = 0.80; p = 4.9x10^-5^); rs2409747/*XKR6* (HR = 1.37; p = 5.4x10^-5^); rs114157400/*BANK1* (HR = 1.62; p = 8.4x10^-5^); and rs72717025/*FCGR2A* (HR = 1.84; p = 9.6x10^-5^) (**Fig 2A; Table 2**). These SNPs are novel candidate SNPs with suggestive evidence and require further confirmation studies to rule out false positive discoveries. The Kaplan-Meier plots of three SNPs (rs2409747/*XKR6*, rs117561283/*IFNG*, and rs8013918/*FOS* discovered in time-to-tTGA analysis are shown in **Fig 2C**.

**Table 2:** Novel associations with celiac disease or tissue transglutaminase autoantibody (tTGA) positivity (p<10^−4^).

SNP^a^	CHR	BP	MAF	HR CD	P-value^b^ CD	HR tTGA	P-value^b^ tTGA	# SNPS (P<10^−4^)	Nearby Gene
rs72704176	1	153692482	0.02	2.26	**7.42x10**^**-5**^	1.31	0.085	1	ASH1L
rs3771689	2	159930048	0.14	0.56	**9.27x10**^**-5**^	0.85	0.037	2	BAZ2B
rs13014907	2	185781851	0.01	2.46	**5.99x10**^**-5**^	1.45	0.050	1	ZNF804A
rs11739460	5	149685099	0.40	1.41	**2.88x10**^**-5**^	1.05	0.319	2	TCOF1
rs77532435	7	50641412	0.03	2.05	**4.82x10**^**-5**^	1.38	0.015	1	GRB10
rs6967298	7	69652445	0.19	0.61	**9.42x10**^**-5**^	0.90	0.137	1	AUTS2
rs61751041	7	107381421	0.02	2.23	**9.76x10**^**-5**^	1.61	0.002	1	LAMB1
rs72717025	1	159736883	0.02	1.41	0.207	1.84	**9.61x10**^**-5**^	1	FCGR2A
rs114157400	4	103154484	0.04	1.71	0.003	1.62	**8.43x10**^**-5**^	2	BANK1
rs2409747	8	11115872	0.11	1.58	**9.28x10**^**-5**^	1.37	**5.37x10**^**-5**^	1	XKR6
rs117561283	12	66732860	0.03	1.96	0.002	1.81	**2.14x10**^**-5**^	1	IFNG
rs8013918	14	74779319	0.45	0.85	0.066	0.80	**4.91x10**^**-5**^	1	FOS

CHR: Chromosome; BP: Base Pair Position (NCBI 36.3); MAF: Minor Allele Frequency; HRCD: Hazard Ratio in celiac disease analysis; HRtTGA: Hazard Ratio in tTGA analysis. P-values < 10^−4^ are highlighted in bold

^a^The data for the SNP with smallest p-value is presented from each region.

^b^HRs and p-value adjusted for family history of celiac disease, HLA-DR-DQ genotype, gender, *HLA-DPB1*, population stratification (ancestral heterogeneity) and country of residence (as strata).

### SNPs associated with progression to celiac disease outside of the known celiac disease regions

In a similar analysis using time-to-celiac disease with all SNPs, no SNP reached the Bonferroni-corrected p<3.7x10^-7^ significance threshold but 10 SNPs outside of the known celiac disease regions reached the suggestive threshold (p<10^−4^) (**Fig 2B**). These SNPs mapped to 8 different regions: rs11739460/*TCOF1* (HR = 1.41; p = 2.9x10^-5^); rs77532435/*GRB10* (HR = 2.05; p = 4.8x10^-5^); rs13014907/*ZNF804A* (HR = 2.46; p = 6.0x10^-5^); rs72704176/*ASH1L* (HR = 2.26; p = 7.4x10^-5^); rs3771689/*BAZ2B* (HR = 0.56; p = 9.3x10^-5^); rs2409747/*XKR6* (HR = 1.58; p = 9.3x10^-5^); rs6967298/*AUTS2* (HR = 0.61; p = 9.4x10^-5^); and rs61751041/*LAMB1* (HR = 2.23; p = 9.8 x10^-5^) (**Table 2**). The Kaplan-Meier plots of three novel SNPs (rs13014907/*ZNF804A*, rs11739460/*TCOF1*, and rs77532435/*GRB10* discovered in time-to-celiac disease analysis are shown in **Fig 2D**.

### Country-specific genetic factors associated with progression to celiac disease

**Fig 3:**
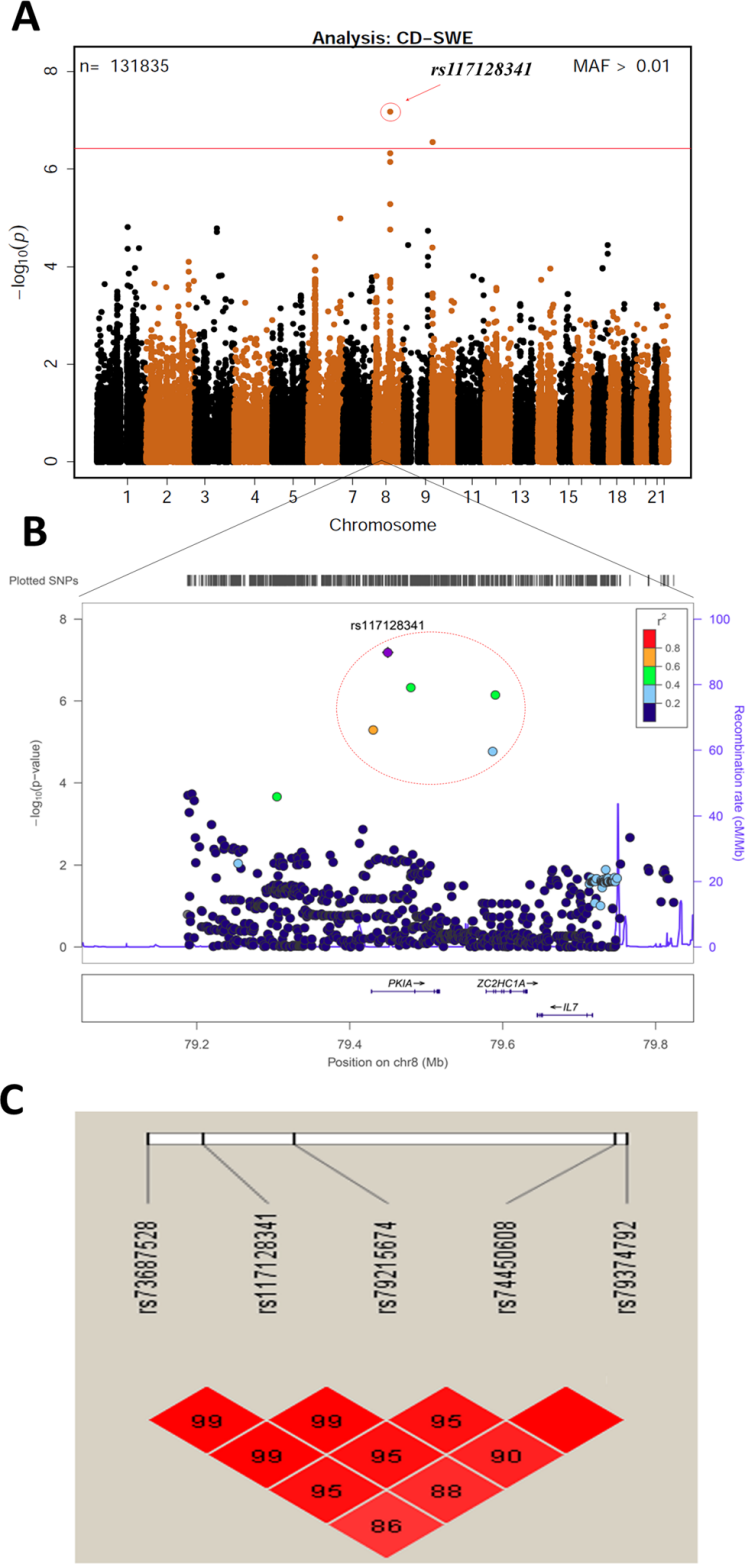
Associations with risk of celiac disease in the Swedish population. **A:** Manhattan plot of 133620 SNPs with MAF>0.01, displaying the *P*-values on the −log_10_ scale for the SNPs associated with celiac disease in the Swedish TEDDY population. **B:** Regional association plots at the *PKIA* locus generated by LocusZoom, showing the significance of association and the recombination rate. Colors represent HapMap CEU linkage disequilibrium r^2^ values with the most significantly associated SNP (rs117128341; shown in purple). **C:** Pairwise LD plot for five SNPs in the region of *PKIA*. The five most significant SNPs from this region are in high LD with each other.

**Fig 4:**
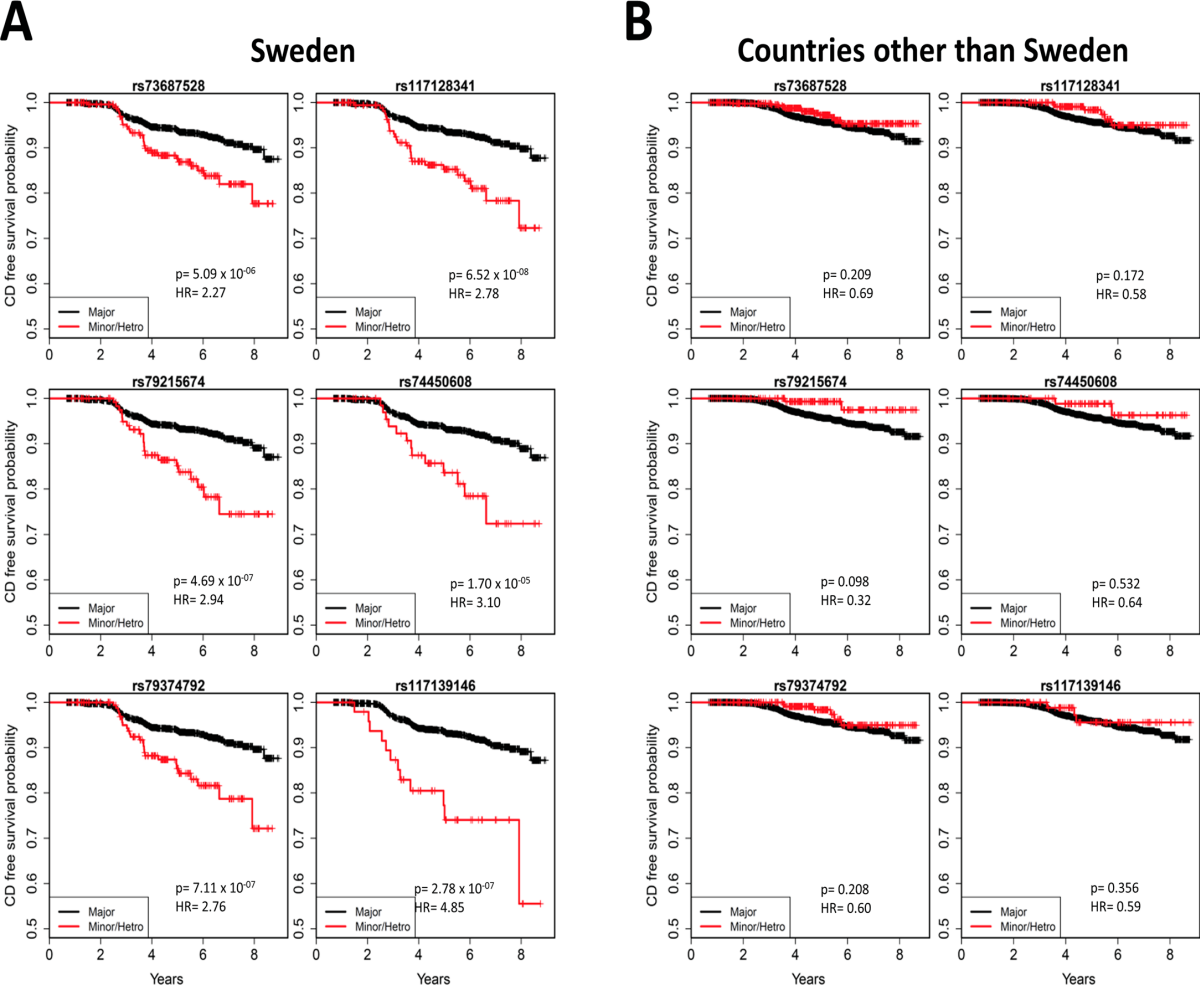
Country-specific associations with risk of celiac disease. Kaplan-Meier plots of five SNPs mapped to the *PKIA* region and one SNP mapped to the *PFKFB3* region, in the Swedish TEDDY population (**A**) and in the other TEDDY countries (**B**). Kaplan-Meier plots clearly indicate country-specific differences. HRs and p-values are calculated using three possible genotypes and adjusted for family history of celiac disease, HLA-DR-DQ genotype, gender, *HLA-DPB1* and population stratification (ancestral heterogeneity).

To explore country-specific genetic factors, the data with all 133,620 SNPs were analysed for each country. In the analysis of celiac disease risk among the Swedish participants, SNPs reached the Bonferroni-corrected p<3.7x10^-7^ significance threshold in two regions: 8q21.1 and 10p15 (**Fig 3**). The SNP with the smallest p-value in the 8q21.1 region was rs117128341 (p = 6.52x10^-8^, HR = 2.78, MAF = 0.04), a SNP in the intragenic region of the protein kinase inhibitor alpha (*PKIA*) gene. The other two nearby genes in this region are *ZC2HC1A* and *IL7*. The SNP with the smallest p-value in the chromosome 10p15 region was rs117139146 (p = 2.78x10^-7^, HR = 4.85, MAF = 0.014). A nearby gene in this region is *PFKFB3*, which was previously shown to be associated with celiac disease. Five SNPs with p<10^−4^ map to the *PKIA* region, and one SNP maps to the *PFKFB3* region (**Table 3)**. In the separate analyses of two other countries, the US and Finland, none of the six SNPs reached the significance threshold. The analysis in Germany was not conducted due to small sample size. Kaplan-Meier plots of these six SNPs for different countries (**Fig 4)** clearly indicated country-specific differences. The associations of these six SNPs with celiac disease in Sweden and the other three countries are listed in **Table 3**. There was no evidence of difference between US and Finland, therefore we combined US, Finland and Germany together to compare with Sweden in the analysis of interaction. A Cox proportional hazards model with an interaction term of the SNP with country was used to compare the effects of these six SNPs between Sweden and other countries, adjusting for country (Sweden vs other), gender, HLA-DPB1 genotype, HLA-DR-DQ genotype, family history of celiac disease, and population stratification. The analysis shows that the effect of any of these SNPs in Sweden is statistically different (p<0.05) from the effect in the other countries (rs73687528: p<0.001; rs117128341: p<0.001; rs79215674: p = 0.002; rs74450608: p = 0.029; rs79374792: p<0.001; rs117139146: p = 0.001).

**Table 3:** Six SNPs from two genomic regions significantly associated with celiac disease in Sweden. Five SNPs mapped to *PKIA* region and one SNP mapped *PFKFB3* region.

				Sweden		Other TEDDY countries than Sweden^c^		Sweden *vs* other TEDDY countries^c^
SNP	CHR	BP	MAF	HR^a^	p^a^	HR^a^	p^a^	p^b^
rs73687528	8	79593304	0.064	2.27	5.09x10^-6^	0.69	0.209	<0.001
rs117128341	8	79612329	0.040	2.78	6.52x10^-8^	0.58	0.172	<0.001
rs79215674	8	79642439	0.027	2.94	4.69x10^-7^	0.32	0.098	0.002
rs74450608	8	79749568	0.016	3.10	1.70x10^-5^	0.64	0.532	0.029
rs79374792	8	79753034	0.040	2.76	7.11x10^-7^	0.60	0.208	<0.001
rs117139146	10	6240562	0.014	4.85	2.78x10^-7^	0.59	0.356	0.001

CHR: Chromosome; BP: Base Pair Position (NCBI 36.3); MAF: Minor Allele Frequency; HR: Hazard Ratio.

^a^HRs and p-value adjusted for family history of celiac disease, HLA-DR-DQ genotype, gender, *HLA-DPB1* and population stratification (ancestral heterogeneity).

^b^P-value of testing the hypothesis that the effects of the SNP are the same between Sweden and other countries from a Cox model with adjustment for family history of celiac disease, HLA-DR-DQ genotype, gender, *HLA-DPB1*, population stratification (ancestral heterogeneity) and country of residence (Sweden vs. other).

^c^Other participating countries of TEDDY are Germany, Finland and the US.

### Country-specific associations with progression to tTGA

In time-to-persistent tTGA analysis among subjects from Sweden, none of the SNPs reached the Bonferroni-corrected p<3.7x10^-7^ significance threshold, however, 9 SNPs in 9 different genomic regions had p<10^−4^ (**S5 Table**). One such SNP was rs117139146 in the region of *PFKFB3* (p = 7.34x10^-5^, HR = 2.79, MAF = 0.014).

## Discussion

HLA-DR3-DQ2.5 and DR4-DQ8 are known as the most important genetic risk factors for celiac disease; however, these two haplotypes only account for part of the genetic risk. Recently, we demonstrated that HLA can be used to assess the risk of celiac disease using the large prospective TEDDY cohort [13]. This previous study clearly demonstrated an HLA gene dose effect of HLA-DR3-DQ2.5 on the risk of celiac disease autoimmunity was doubled among heterozygotes (HR = 2.09) but was a near 6-fold increased among homozygotes (HR = 5.70) as compared to children carrying the lowest-risk genotype DR4-DQ8 [13]. However, another finding of importance from this study was the difference in incidence of celiac disease between the participating countries which could not be attributed to HLA suggesting that environmental factors or other genes could contribute to the disease risk. In the current study, we used the same cohort to assess the association of non-HLA genes to the progression to tTGA in addition to progression to celiac disease in early childhood. A strength of this study includes the prospective nature of the study cohort that time-to-events analyses can be conducted, looking specifically in this case for genetic factors that could be related to the *early* development of celiac disease. Genetic studies have been traditionally done using cross-sectional case/control study design, with populations of individuals with celiac disease who have an unknown age of actual onset of autoimmunity. We know that the rate of seroconversion and subsequent development of celiac disease is high in childhood, and suspect that the yearly incidence slows down some time in adulthood. It is therefore possible that the genes involved in early onset celiac disease may be different from those involved in adult (or late) onset celiac disease. However, it may not be feasible to perform a prospective cohort study in at-risk adults due to the presumed decline in incidence.

In the first stage of analyses, we only considered the 48 SNPs previously reported to be associated with celiac disease and only one SNP was significant after Bonferroni correction. However, confirmatory evidence (p<10^−4^) was found for SNPs in five regions previously reported to be associated with celiac disease (*TAGAP*, *IL18R1*, *RGS21*, *PLEK*, and *CCR9*). The HRs estimated in this prospective cohort (HR = 1.40–2.64) are generally much higher than the odds ratios (OR) estimated in the case control studies (OR = 1.12–1.36). The *TAGAP* gene encodes a member of the Rho GTPase-activator protein superfamily involved in T cell activation and co-regulation with *IL-2*, which has been previously associated with several autoimmune diseases, including rheumatoid arthritis [19], celiac disease [20], and multiple sclerosis[21]. *IL18R1* is part of the cytokine receptor cluster on chromosome 2q12 which encodes for the receptors of IL18; a cytokine involved in IFN-gamma synthesis and its mRNA expression is upregulated in active patients with celiac disease [22]. Both genes play roles in the immune response and are therefore rational candidates for conferring risk in an autoimmune disease such as celiac disease.

The development of tTGA usually appears before the clinical onset of celiac disease and often represents the earliest stage of autoimmunity, signifying a breakdown in tolerance. The specificity of tTGA is high such that negative testing will almost certainly rule out celiac disease. However the positive predictive value of the antibody especially in screened cohorts is lower, between 70–83%[23], and some may even be transient[24]. Nevertheless, individuals with only positive tTGA (even without evidence of villous atrophy) should not be disregarded. Positive tTGA is an independent predictor of reduced bone mineral density[25], growth[26] and mortality[27] and has been demonstrated to progress to celiac disease. In addition, many individuals with positive celiac disease serology but normal villous morphology have been shown to subsequently develop celiac disease in subsequent follow-up[28].

Although HLA genes are known to contribute to the development of tTGA, the contribution of non-HLA genes to the development tTGA and its role in early childhood celiac autoimmunity is still not well characterized. This study suggests that there are a number of non-HLA genes potentially implicated in the development of tTGA, and that there is overlap between genes involved in both tTGA and celiac disease development. For example, *CTLA4* and *LPP* are implicated in both celiac disease and tTGA development, although the association with tTGA appears to be stronger than with celiac disease. On the other hand, association evidence for *RGS21*, *IL18R1*, *PLEK*, *CCR9*, *TAGAP* was only found for celiac disease (**Table 1**).

Our recent studies on HLA class II genes in the TEDDY cohort also demonstrated that the Swedish participants were at an increased risk for early celiac disease as compared to other participating countries in TEDDY when adjusted for previously known risk factors[13]. We hypothesized that this increased risk was due to variations in exposures to environmental factors. However, an alternative explanation is that there could be genetic differences outside of the HLA-DR-DQ genes between Sweden and other countries which, in part, may account for differences in disease incidences. The current study tested this hypothesis and found two regions (chromosomes 8q21.1 and 10p15) with Bonferroni-corrected significance evidence in the Swedish dataset, but not in the other three countries.

The chromosome 8q21.1 region is a novel genomic interval associated with celiac disease in Sweden and contains five SNPs with strong evidence (1.7x10^-5^>p>6.5x10^-8^). It is near the *PKIA* gene which encodes an extremely potent competitive inhibitor of cAMP-dependent protein kinase. It has been previously reported that intestinal *PKIA* gene expression was increased among patients with untreated celiac disease [29]. Another study suggests a potential role of cAMP-dependent protein kinase-A activation in the TNF-alpha production by gliadin-derived peptides in intestinal epithelial cells [30].

One SNP (rs117139146) located in the intragenic region of chromosome 10p15 encoding for *PFKFB3* (6-phosphofructo-2-kinase/fructose-2,6-biphosphatase 3) was initially identified as being associated with celiac disease through the 1000 Genomes Project using the ImmunoChip in 2012 [8]. The 440kB region between *PFKFB3* and Protein Kinase C Theta (*PRKCQ*) has been reported in a meta-analysis to identify rheumatoid arthritis (RA) risk loci in European populations [31], and also has been shown to be associated with T1D [32]. In a meta-analysis of Dutch and UK data sets, shared association with this *PFKFB3/PRKCQ* region was observed in both RA and celiac disease [33]. In a study of North Americans, this region was suggestive of an association with celiac disease, but did not reach significance[9].

Recently, two other studies have also shown region-specific associations observed in celiac disease. The prevalence of tTGA and celiac disease is lower in Russian Karelia than in Finland, which may be associated with a lower economic status and inferior hygienic environment.[34] Also, discrepancy of celiac disease autoimmunity between Swedish and Danish T1D cohorts suggests that regional variations in comorbidity of celiac disease in T1D is caused by difference in exposure to environmental factors. [35]. Country-specific associations have also been observed in other autoimmune diseases. For example, *PADI4* was the first non-HLA genetic risk factor known to be associated with RA, in a Japanese population[36]. However, in Spanish, Swedish and UK populations, *PADI4* polymorphisms were not associated with RA [37,38]. Gene-environment interactions probably are more important in diseases where the ingestion of a particular type of food is required to maintain or trigger the disease. Recently, it has been shown in Australia that infants of Asian-born parents are at increased risk of peanut allergy compared to infants with parents migrating from other countries, suggesting gene-environment interactions are important in food allergy [39].

It is worth noting that our inference is based on a subset of SNPs included in the Illumina ImmunoChip genotyping platform. Also, this analysis lacks power to confirm or discover genetic factors with small effect size due to the limited number of events and short follow-up time. None of the SNPs reached the Bonferroni-corrected significance threshold in the entire dataset, although two regions reached the significance level in the Sweden population that has a higher incidence rate of celiac disease. Analyses of the TEDDY cohort with longer follow-up and more events will likely provide more robust evidence for the newly suggested and previously identified genetic factors. However, current age of our cohort allows the analysis of factors involved in the earliest development of celiac autoimmunity and celiac disease, which may vary from celiac disease that develops in an older population. Our study also highlights the necessity of having another large prospective cohort like TEDDY to fully elucidate the genetic mechanism of celiac disease. It is also worth noting that the HRs presented in this analysis are based on a population of children enriched for the high-risk celiac HLAs, and the findings may not be generalizable to the general population. TEDDY is the largest and most intensive study focusing on the genetic and environmental factors as well as gene-environment interactions for diabetes and celiac disease [12]. The current study has not explored genetic factors in the context of environmental exposure data and we believe that future integrated analyses of gene-environment interactions will allow us to reveal the underlying molecular mechanism of the disease.

## Material and Methods

### Material

**Fig 5:**
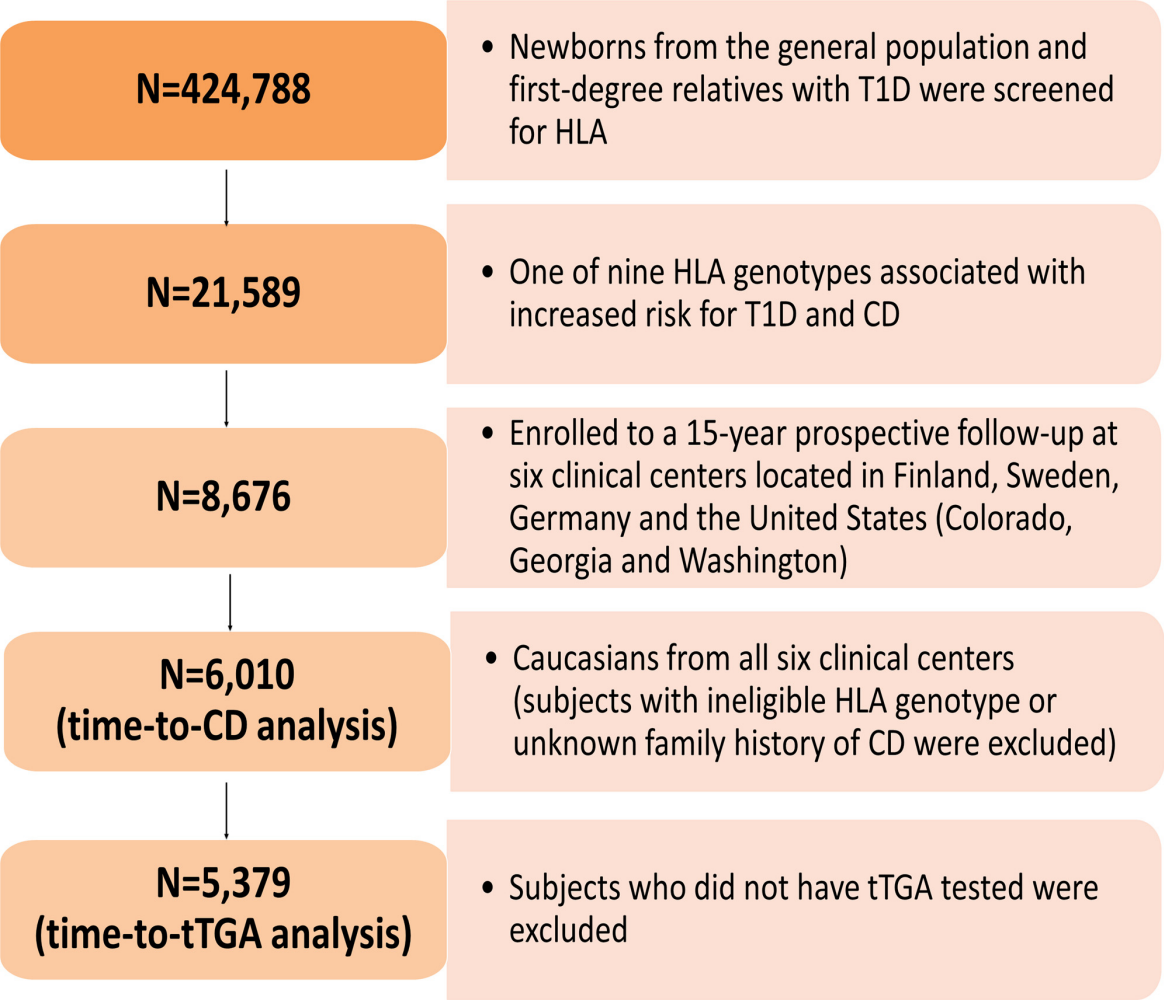
Flow chart of study participants. The Environmental Determinants of Diabetes in the Young (TEDDY) is an international multicenter study that screened over 420,000 newborns from the general population in four different countries. The present study genotyped 195,806 SNPs on ImmunoChip in 6,010 TEDDY children to identify potential genetic factors responsible for the development of CD and country-specific differences in genetic predisposition. As shown in flow chart, a total of 6,010 subjects were included in the analysis of time-to-CD, and 5379 subjects were included in the analysis of time-to-tTGA.

A total of 424,788 newborns from both the general population and first-degree relatives with T1D were screened for specific HLA genotypes. Of these, 21,589 had one of the nine HLA genotypes associated with increased risk for T1D and celiac disease (**S1 Table**) and 8,676 eligible children were enrolled to a 15-year prospective follow-up[40] (**Fig 5).**

The Environmental Determinants of Diabetes in the Young (TEDDY) is a prospective cohort study with the primary goal to identify environmental causes of T1D. This study was performed according to the principles of the Declaration of Helsinki. Written informed consent was obtained for all study participants from a parent or legal guardian. The TEDDY study was approved by local Institutional Review Boards at 6 clinical research centers (3 in the United States and 3 in Europe): University of Colorado Health Science Center, Georgia Regents University, Pacific Northwest Diabetes Research Institute, Turku University Hospital (Finland), Institute of Diabetes Research (Germany), and Lund University (Sweden). The study is also monitored by an external evaluation committee formed by the National Institutes of Health.

### Assessment of tissue transglutaminase autoantibodies (tTGA)

Sera were measured for tTGA using radioligand binding assays in two laboratories, IgA-tTGA assay at the Barbara Davis Center for Childhood Diabetes for the US samples and IgA-tTGA and IgG-tTGA assay at the University of Bristol for European samples. All positive US samples were also assayed by the Bristol lab. Levels of tTGA were expressed in arbitrary units derived from a standard curve and were considered to be positive if the value was ≥1.3 units. The inter-assay coefficient of variation was 22% at both 6 units and 20 units.

### Study outcomes

Persistent tTGA was defined as having two consecutive positive tTGA tests (as measured by the Bristol laboratory) taken at least three months apart. Children meeting this criterion were referred to a pediatric gastroenterologist for a clinical evaluation for celiac disease. Celiac disease was defined as having an intestinal biopsy showing a Marsh score of 2 or greater by original Marsh criteria [41]. Children who had persistent tTGA with a mean level of >100 units in two consecutive samples but had no intestinal biopsy data, were also considered as having celiac disease for the purpose of this study [13].

### Single-nucleotide polymorphism (SNP) analysis by ImmunoChip

SNPs were genotyped by the Center for Public Health Genomics at University of Virginia, using the Illumina ImmunoChip Infinium array. The ImmunoChip is a custom genotyping array of 195,806 SNPs selected from 186 regions associated with 12 autoimmune diseases. Genotype calling and quality control steps were applied to the dataset: (1) individuals with low call rate (<95%), or discordance with reported gender and prior genotyping were not considered in the analysis, (2) SNP markers with low call rates (<95%) were excluded, and (3) markers with allele distributions strongly deviating from Hardy-Weinberg equilibrium (HWE) in controls (p<10^−6^) were discarded (except for chromosome 6 due to HLA eligibility requirements). This resulted in a total of 7,023 subjects with genotype data on 176,586 SNPs.

### Statistical analysis

The time-to-persistent tTGA and the time-to-celiac disease were the two primary outcomes analysed in this study. The time-to-persistent tTGA was defined as the age when the sample for the first tTGA positive test was collected, and the right-censored time was the age when the participant’s last blood sample was collected for testing of tTGA. The time-to-celiac disease was the child’s age at the time of biopsy for the diagnosis of celiac disease, or the age of the first high-level tTGA result (defined as ≥100 units). The right-censored time was the age of the last TEDDY clinic visit that was confirmed to be celiac disease-free. Cox proportional hazards modelling was used to analyse the effect of each individual SNP (by genotype 0,1,2) on the outcome, after adjusting for country (as strata), gender, HLA-DPB1 genotype, HLA-DR-DQ genotype (e.g., DR3-DQ2/DR3-DQ2, DR3-DQ2/X, DR4-DQ8/DR4-DQ8 and Other), family history (first-degree relative) of celiac disease, and principal components to account for population stratification (ancestral heterogeneity). The Cox models were fitted using the “survival” package in R [42]. The first four principal components were used in these analyses, calculated from the SNP data using the SNPRelate software [43]. As the majority of the subjects were Caucasians, and to reduce population stratification further, subjects from other races were excluded and analyses were restricted to the 6,258 Caucasians from all six clinical centers.

Subject exclusion included those who had either an ineligible HLA genotype or unknown family history (among first-degree relatives) of celiac disease. Further, subjects who did not have tTGA tested were also excluded in the analysis of time-to-persistent tTGA. As a result, 6,010 subjects were included in the analysis of time-to- celiac disease, and 5,379 subjects were included in the analysis of time-to- persistent tTGA (**Fig 5**). Among the 6,010 subjects included in the analysis of celiac disease, the median follow-up time was 5 years (interquartile range: 3.75–6.44 years). Whereas, among the 5,379 subjects included in the analysis of persistent tTGA, the median follow-up time was 5.18 years with an interquartile range of 4.04–6.54 years. During the follow-up, a total of 703 subjects developed persistent tTGA (US: 191 out of 1785; Finland: 167 out of 1414; Germany: 37 out of 328; Sweden: 308 out of 1852) and a total of 288 subjects were considered as having celiac disease (US: 83 out of 2028; Finland: 53 out of 1551; Germany: 9 out of 399; Sweden: 143 out of 2032). The characteristics of TEDDY participants in the analyses of persistent tTGA (**S2 Table**) and celiac disease (**S3 Table**) are provided in the Supplementary material.

From the 176,586 SNPs that passed quality control filters, the analysis focused on those 133,620 with minor allele frequencies of at least 0.01; thus, statistical significance for any single SNP required a Bonferoni-corrected p<3.7x10^-7^. This is a highly stringent threshold as a large number of SNPs are in linkage disequilibrium which should reduce the total number of independent tests. For the analyses of the 48 candidate SNPs that have been identified in previous studies, we considered p<10^−3^ as suggestive evidence for confirmation (p = 0.05/48 = 10^−3^). For the analyses of the 48 candidate regions, we considered p<10^−4^ as suggestive evidence for confirmation as multiple SNPs are tested in each region and the SNPs are in high linkage disequilibrium.

All analyses were performed using R 2.15.1. A web-based plotting tool *locuszoom* [44] was used to plot HapMap CEU linkage disequilibrium r^2^ values for additional SNPs in the candidate SNP regions.

## Supporting Information

S1 Table Enrolled HLA genotypes in the TEDDY study. (PDF)

S2 Table Characteristics for Celiac Disease Autoimmunity. (PDF)

S3 Table Characteristics for Celiac Disease. (PDF)

S4 Table Analysis of reported celiac disease risk variants. (PDF)

S5 Table Analysis of celiac disease risk variants only in Sweden population. (PDF)

S6 Table Risk Variants that have been reported but are not on the IChip. (PDF)

S1 Fig SNPs associated with tTGA risk. (PDF)

S2 Fig SNPs associated with celiac disease risk. (PDF)

S1 Text TEDDY Study Acknowledgments; All TEDDY sites and investigators. (PDF)

## Abbreviations

RA: rheumatoid arthritis
SNP: Single-nucleotide polymorphism
TEDDY: The Environmental Determinants of Diabetes in the Young
tTGA: tissue transglutaminase autoantibodies
T1D: type 1 diabetes
