# Supplementary material for: Identification of Non-HLA Genes Associated with Celiac Disease and Country-Specific Differences in a Large, International Pediatric Cohort
Source: PLoS One. 2016 Mar 25;11(3):e0152476. doi: 10.1371/journal.pone.0152476 (PMC4807782; doi:10.1371/journal.pone.0152476)
Supplement: S1 Table — (PDF) [file pone.0152476.s001.pdf]

**S1 Table: Enrolled HLA genotypes in the TEDDY study**

| LETTER CODE | FULL GENOTYPE | ABBREV. |
|---|---|---|
| A | DRB1*04-DQA1*03-DQB1*03:02/DRB1*03-DQA1*05-DQB1*02:01 | DR 3/4 |
| B | DRB1*04-DQA1*03-DQB1*03:02/DRB1*04-DQA1*03-DQB1*03:02 | DR 4/4 |
| C | DRB1*04-DQA1*03-DQB1*03:02/DRB1*08-DQA1*04-DQB1*04:02 | DR 4/8 |
| D | DRB1*03-DQA1*05-DQB1*02:01/DRB1*03-DQA1*05-DQB1*02:01 | DR 3/3 |
| E | DRB1*04-DQA1*03-DQB1*03:02/DRB1*04-DQA1*03-DQB1*02:02 | DR 4/4* |
| F | DRB1*04-DQA1*03-DQB1*03:02/DRB1*01-DQA1*01-DQB1*05:01 | DR 4/1 |
| G | DRB1*04-DQA1*03-DQB1*03:02/DRB1*13-DQA1*01-DQB1*06:04 | DR 4/13 |
| I | DRB1*04-DQA1*03-DQB1*03:02/DRB1*09-DQA1*03-DQB1*03:03 | DR 4/9 |
| J | DRB1*03-DQA1*05-DQB1*02:01/DRB1*09-DQA1*03-DQB1*03:03 | DR 3/9 |

Note: genotypes A, B, C and D confer general population eligibility but exclude DRB1*0403. Genotypes A through J confer eligiblity to a first-degree relative of a diabetic patient. where DQB1*03:02 is noted, either this allele or DQB1*03:04 is allowed. see Hagopian et al 2011 Pediatric Diabetes 17:733-43.
