# Supplementary material for: Identification of Non-HLA Genes Associated with Celiac Disease and Country-Specific Differences in a Large, International Pediatric Cohort
Source: PLoS One. 2016 Mar 25;11(3):e0152476. doi: 10.1371/journal.pone.0152476 (PMC4807782; doi:10.1371/journal.pone.0152476)
Supplement: S2 Table — (PDF) [file pone.0152476.s002.pdf]

S2 Table. Characteristics for Celiac Disease Autoimmunity

	703 subjects Developed tTGA<br>mean (SD) or N (%)	4676 subjects did not develop tTGA<br>mean (SD) or N (%)	HR (95%CI) [a]	p [a]
Age at first tTG+ visit or most recent visit (years)	3.06 (SD=1.33)	4.84 (SD=1.67)		
Country				
US	191 (27.2)	1594 (34.1)		
Finland	167 (23.7)	1247 (26.7)		
Germany	37 (5.3)	291 (6.2)		
Sweden	308 (43.8)	1544 (33.0)		
Family History of celiac disease				
Yes	39 (5.6)	102 (2.2)	1.68 (1.21, 2.32)	0.002
HLA-DR, -DQ genotype				< 0.001[c]
DR3-DQ2/DR3-DQ2	312 (44.4)	823 (17.6)	3.46 (2.60, 4.60)	
DR3-DQ2/X	272 (38.7)	1879 (40.2)	1.36 (1.06, 1.75)	
DR4-DQ8/DR4-DQ8	93 (13.2)	943 (20.2)	1	
Other	26 (3.7)	1031 (22.0)	0.25 (0.16, 0.38)	
HLA DPB1				
0	300 (42.7)	1537 (32.9)		
1	319 (45.4)	2279 (48.7)	0.81 (0.73, 0.91)[b]	< 0.001
2	84 (11.9)	860 (18.4)		
Gender				
Female	421 (59.9)	2215 (47.4)	1.71 (1.47, 1.99)	< 0.001

[a]HRs and p-values adjusted for family history of celiac disease, HLA-DR_DQ genotype, gender, HLA DPB1, population stratification (ancestral heterogeneity) and country of residence (as strata).
[b] HR for an increase of one copy of minor allele.
[C] P-value from the test of no different effects among the four HLA-DR-DQ genotype groups.
