# Supplementary material for: Identification of Non-HLA Genes Associated with Celiac Disease and Country-Specific Differences in a Large, International Pediatric Cohort
Source: PLoS One. 2016 Mar 25;11(3):e0152476. doi: 10.1371/journal.pone.0152476 (PMC4807782; doi:10.1371/journal.pone.0152476)
Supplement: S3 Table — (PDF) [file pone.0152476.s003.pdf]

S3 Table. Characteristics for Celiac Disease

	288 subjects developed celiac disease<br>mean (SD) or N (%)	5722 subjects did not develop tTGA<br>mean (SD) or N (%)	HR (95%CI) [a]	p [a]
Age at celiac disease diagnosis visit or most recent visit (years)	3.87 (SD=1.40)	4.88 (SD=1.97)		
Country				
US	83 (28.8)	1945 (34.0)		
Finland	53 (18.4)	1498 (26.2)		
Germany	9 (3.1)	390 (6.8)		
Sweden	143 (49.7)	1889 (33.0)		
Family History of celiac disease				
Yes	25 (8.7)	123 (2.2)	2.67 (1.75, 4.06)	< 0.001
HLA-DR, -DQ genotype				< 0.001[c]
DR3-DQ2/DR3-DQ2	154 (53.5)	1120 (19.6)	3.19 (2.07, 4.92)	
DR3-DQ2/X	90 (31.3)	2298 (40.2)	0.96 (0.64, 1.42)	
DR4-DQ8/DR4-DQ8	40 (13.9)	1118 (19.5)	1	
Other	4 (1.4)	1186 (20.7)	0.10 (0.04, 0.28)	
HLA DPB1				
0	132 (45.8)	1936 (33.8)		
1	118 (41.0)	2762 (48.3)	0.81 (0.68, 0.97)[b]	0.021
2	38 (13.2)	1024 (17.9)		
Gender				
Female	187 (64.9)	2756 (48.2)	2.15 (1.69, 2.75)	< 0.001

[a]HRs and p-values adjusted for family history of celiac disease, HLA-DR_DQ genotype, gender, HLA DPB1, population stratification (ancestral heterogeneity) and country of residence (as strata).
[b] HR for an increase of one copy of minor allele.
[C] P-value from the test of no different effects among the four HLA-DR-DQ genotype groups.
