# Supplementary material for: Identification of Non-HLA Genes Associated with Celiac Disease and Country-Specific Differences in a Large, International Pediatric Cohort
Source: PLoS One. 2016 Mar 25;11(3):e0152476. doi: 10.1371/journal.pone.0152476 (PMC4807782; doi:10.1371/journal.pone.0152476)
Supplement: S4 Table — (PDF) [file pone.0152476.s004.pdf]

S4 Table. Analysis of reported celiac disease risk variants

SNPs	Region	Chr_id	Reported Genes	MAF	HR-CD	P-CD	HR-TG	P-TG
rs3748816	1p36.32	1	TNFRSF14, MMEL1	0.335	1.05	0.6200	1.004	0.9386
rs12727642	1p36.23	1	PARK7, TNFRSF9	0.199	0.97	0.7957	0.981	0.7744
rs10903122	1p36.11	1	RUNX3	0.502	0.88	0.1295	1.010	0.8502
rs6691768	1p31.3	1	NFIA	0.388	0.93	0.4332	0.943	0.2906
rs864537	1q24.2	1	CD247	0.369	0.88	0.1380	0.869	0.0133
rs859637	1q24.3	1	FASLG, TNFSF18, TNFSF4	0.499	0.97	0.7405	0.938	0.2265
rs2816316	1q31.2	1	RGS1	0.177	0.81	0.0757	0.859	0.0378
rs296547	1q32.1	1	Intergenic	0.378	1.02	0.8063	1.023	0.6851
rs7579944	2p23.1	2	LBH	0.380	0.95	0.5830	1.033	0.5625
rs13003464	2p16.1	2	REL, AHSA2	0.389	1.05	0.5542	0.949	0.3387
rs1876518	2p14	2	SPRED2	0.425	0.97	0.7254	1.100	0.0774
rs17035378	2p14	2	PLEK	0.279	0.75	0.0037	0.914	0.1437
rs13015714	2q12.1	2	IL1RL1,IL18R1,IL18RAP, SLC9A4	0.239	1.42	0.0001	1.153	0.0201
rs7574865	2q32.3	2	STAT4	0.226	0.97	0.7866	1.140	0.0358
rs4675374	2q33.2	2	CTLA4, ICOS, CD28	0.206	0.92	0.4494	1.111	0.1024
rs13314993	3p22.3	3	CCR4	0.427	1.10	0.2536	0.993	0.8990
rs13098911	3p21.31	3	CCR1, CCR2, CCRL2, CCR3, CCR5, CCR9	0.102	1.29	0.0411	1.124	0.1622
rs6441961	3p21.31	3	CCR1, CCR3	0.300	1.07	0.4552	1.034	0.5642
rs6806528	3p14.1	3	FRMD4B	0.096	1.44	0.0041	1.131	0.1631
rs11712165	3q13.33	3	CD80, KTELC1	0.394	1.17	0.0637	1.120	0.0352
rs17810546	3q25.33	3	IL12A	0.122	1.10	0.4453	0.970	0.7102
rs10936599	3q26.2	3	Intergenic	0.257	1.10	0.3025	1.147	0.0224
rs1464510	3q28	3	LPP	0.473	1.28	0.0023	1.163	0.0043
rs13151961	4q27	4	IL2, IL21	0.148	0.84	0.1640	0.975	0.7382
rs6822844	4q27	4	KIAA1109, ADAD1, IL2, IL21	0.152	0.86	0.1975	0.982	0.8048
rs1033180	6p25.3	6	IRF4	0.092	1.06	0.6929	0.955	0.6203
rs424232	6p21.32	6	HLA	0.430	0.92	0.6553	0.950	0.6502
rs2187668	6p21.32	6	HLA-DQA1, HLA-DQB1	0.409	1.39	0.6185	0.936	0.8706
rs10806425	6q15	6	BACH2, MAP3K7	0.357	1.05	0.5825	0.994	0.9091
rs802734	6q22.33	6	PTPRK, THEMIS	0.300	1.21	0.0336	1.124	0.0456
rs2327832	6q23.3	6	TNFAIP3	0.211	1.24	0.0251	1.055	0.4029
rs1738074	6q25.3	6	TAGAP	0.413	1.17	0.0594	1.088	0.1191
rs6974491	7p14.1	7	ELMO1	0.167	1.12	0.2915	1.005	0.9435
rs9792269	8q24.21	8	Intergenic	0.211	0.89	0.2905	0.977	0.7228
rs1953126	9q33.2	9	TRAF1	0.380	1.02	0.8247	1.051	0.3557
rs1250552	10q22.3	10	ZMIZ1	0.498	1.15	0.0975	1.101	0.0735
rs10892279	11q23.3	11	DDX6	0.213	0.97	0.7620	1.051	0.4442
rs11221332	11q24.3	11	ETS1	0.226	1.29	0.0062	1.011	0.8669
rs10876993	12q13.3	12	CDK4	0.353	0.84	0.0422	0.955	0.4096
rs653178	12q24.12	12	SH2B3	0.465	1.30	0.0019	1.082	0.1486
rs2762051	13q14.2	13	Intergenic	0.204	1.05	0.6148	0.984	0.8050
rs12928822	16p13.13	16	CIITA, SOCS1, CLEC16A	0.161	1.12	0.2733	0.988	0.8713
rs17760268	17q22	17	ANKFN1, NOG	0.067	1.00	0.9880	1.151	0.1719
rs1893217	18p11.21	18	PTPN2	0.167	1.19	0.0985	0.924	0.2779
rs11203203	21q22.3	21	UBASH3A	0.349	1.10	0.2538	1.131	0.0277
rs4819388	21q22.3	21	ICOSLG	0.262	0.97	0.7777	0.957	0.4765
rs2298428	22q11.21	22	UBE2L3, YDJC	0.226	1.27	0.0124	1.172	0.0110
rs5979785	Xp22.2	X	TLR7, TLR8	0.260	0.93	0.3771	1.002	0.9703

HR-CD: Hazard ratio for celiac disease; P-CD: P-value for celiac disease; HR-TG: Hazard ratio for celiac disease autoimmunity; P-TG: P-value for celiac disease autoimmunity; Highlighted cells represent P<0.05
