# Supplementary material for: Identification of Non-HLA Genes Associated with Celiac Disease and Country-Specific Differences in a Large, International Pediatric Cohort
Source: PLoS One. 2016 Mar 25;11(3):e0152476. doi: 10.1371/journal.pone.0152476 (PMC4807782; doi:10.1371/journal.pone.0152476)
Supplement: S5 Table — (PDF) [file pone.0152476.s005.pdf]

S5 Table. Analysis of celiac disease risk variants only in Sweden population

SNP	CHR	BP	MAF	HR-CD	P-CD	HR-TGA	P-TGA	# SNPS (P<10^{-4})	Nearby Gene
rs115195008	1	195885068	0.024712092	2.71	1.17E-03	2.45	6.03E-05	1	DENND1B
rs12060029	1	210638198	0.04328	2.56	4.15E-05	1.87	3.12E-04	1	TMEM206
rs72704176	1	153692482	0.023752399	3.10	1.52E-05	1.64	2.27E-02	2	ASH1L
rs1829618	2	212141570	0.189230277	1.68	7.84E-05	1.23	3.37E-02	1	ERBB4
rs1163374	3	110423193	0.251279591	1.76	1.60E-05	1.35	1.05E-03	2	MORC1, DPPA2
rs2014777	4	70706332	0.290147153	1.18	2.10E-01	1.43	3.98E-05	1	SULT1D1P
rs6842780	4	54320985	0.463218577	0.65	5.44E-04	0.71	5.37E-05	1	RP11-317M11.1
rs1054091	6	159389500	0.166426743	1.85	1.02E-05	1.37	2.02E-03	1	TAGAP
rs1367730	6	33166092	0.32863772	2.02	6.18E-05	1.50	4.26E-04	1	HLA-DPB1,HLA-DPA2
rs857415	6	14767762	0.424424184	1.39	5.65E-03	1.38	7.97E-05	1	RP11-330A16.1,LOC102724463
**rs117128341**	**8**	**79612329**	**0.039833627**	**2.78**	**6.52E-08**	**1.65**	**1.50E-03**	**5**	**PKIA**
rs10511729	9	23547227	0.304	0.54	3.56E-05	0.85	7.97E-02	1	LOC101929563
rs76554494	9	122430566	0.07662774	2.15	1.84E-05	1.30	6.96E-02	3	MEGF9
**rs117139146**	**10**	**6240562**	**0.013515675**	**4.85**	**2.78E-07**	**2.79**	**7.34E-05**	**1**	**PFKFB3**
rs9423406	10	5343690	0.17234485	1.78	3.98E-05	1.27	2.02E-02	1	AKR1C7P,RPL26P28
rs17038814	12	105774382	0.12893937	1.47	1.87E-02	1.57	5.33E-05	1	RIC8B
rs2037744	12	16191109	0.12571977	1.64	1.48E-03	1.56	5.57E-05	1	DERA,SLC15A5
rs77013804	13	43377822	0.035268714	1.71	4.43E-02	2.09	4.42E-05	1	LACC1
rs9914860	17	72848875	0.464165733	0.60	3.53E-05	0.82	1.88E-02	2	SEPT9
rs2534116	23	88571714	0.25536859	0.44	4.93E-06	0.59	6.14E-06	1	LOC102724150

Regions with P<10^{-4} in either tTGA or celiac disease analysis are presented in this table.
HR-CD: Hazard ratio for celiac disease; P-CD: P-value for celiac disease; HR-TG: Hazard ratio for celiac disease autoimmunity; P-TG: P-value for celiac disease autoimmunity; Highlighted cells represent P<10^{-4}
