# Supplementary material for: Identification of Non-HLA Genes Associated with Celiac Disease and Country-Specific Differences in a Large, International Pediatric Cohort
Source: PLoS One. 2016 Mar 25;11(3):e0152476. doi: 10.1371/journal.pone.0152476 (PMC4807782; doi:10.1371/journal.pone.0152476)
Supplement: S6 Table — (PDF) [file pone.0152476.s006.pdf]

S6 Table. Risk Variants that have been reported but are not on the IChip

SNPs	Region	Chr_id	Chr_pos	Reported Genes.	Strongest SNP Risk Allele	Pvalue mlog
rs1020388	5q11.2	5	56264200	ANKRD55	rs1020388-?	6.523
rs1032355	4q23	4	99618739	RG9MTD2, C4orf17, MTTP	rs1032355-C	5.301
rs10439884	21p11.1	21	10540506	BAGE2, TPTE, BAGE	rs10439884-A	5.699
rs10886159	10q26.11	10	117854099	EMX2OS, RAB11FIP2, EMX2	rs10886159-C	6.155
rs11984075	7p14.1	7	37397251	ELMO1	rs11984075-G	7.301
rs12734338	1q32.1	1	202500595	PPP1R12B, SYT2, UBE2T	rs12734338-C	6.523
rs13010713	2q31.3	2	181131318	ITGA4, UBE2E3	rs13010713-G	10.301
rs13128441	4p16.2	4	5160662	STK32B	rs13128441-C	5.097
rs157640	20q13.2	20	54798000	DOK5	rs157640-G	5.301
rs1772408	1q23.1	1	159035859	IFI16	rs1772408-?	6.097
rs195656	16q22.2	16	71013581	HYDIN	rs195656-A	5.155
rs1958589	14q13.1	14	34375170	EAPP, SNX6, C14orf147	rs1958589-C	5.398
rs2068824	1q32.1	1	201625537	NAV1	rs2068824-C	5.222
rs2074404	17q21.31	17	46788073	Intergenic	rs2074404-?	6.000
rs2605393	3p22.3	3	36368109	STAC	rs2605393-G	5.222
rs2664156	19q13.33	19	50873907	KLK2, KLK3, KLKP1, KLK4, KLK15	rs2664156-C	5.222
rs4899260	14q24.1	14	68811487	ZFP36L1	rs4899260-A	6.398
rs4911642	22q11.1	22	15473564	CCT8L2, psiTPTE22	rs4911642-C	5.301
rs4930144	11p15.5	11	2027258	IGF2AS, TH, MRPL23, TNNT3, SYT8, ASCL2, TNNI2, LSP1, IGF2, INS, H19	rs4930144-A	5.155
rs917997	2q12.1	2	102454108	IL18RAP, IL18R1, IL1RL1, IL1RL2	rs917997-A	15.000
rs975730	8q24.21	8	128303768	Intergenic	rs975730-?	7.699
