# Supplementary figures and images for: Identification of Non-HLA Genes Associated with Celiac Disease and Country-Specific Differences in a Large, International Pediatric Cohort
Source: PLoS One. 2016 Mar 25;11(3):e0152476. doi: 10.1371/journal.pone.0152476 (PMC4807782; doi:10.1371/journal.pone.0152476)

**S1 Fig: SNPs associated with tTGA risk**

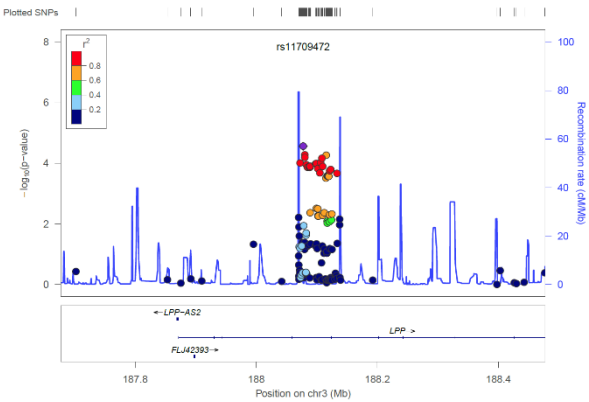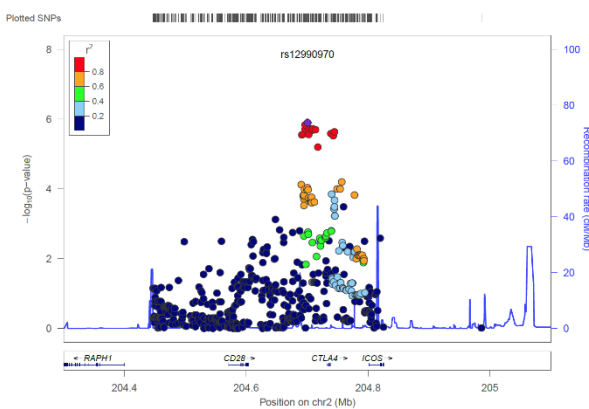

Supplement: S1 Fig — (PDF) [file pone.0152476.s007.pdf]

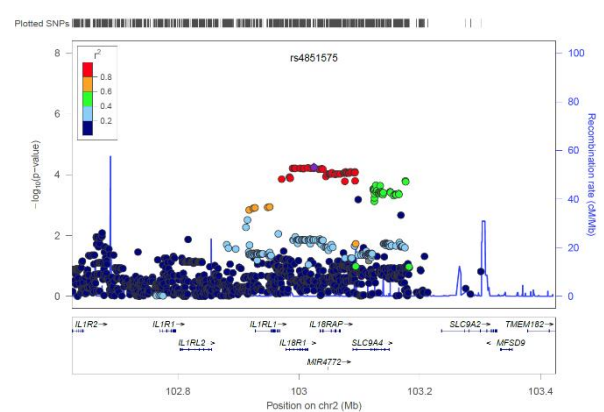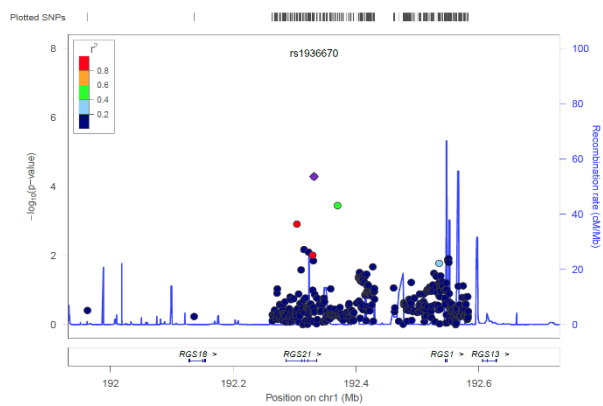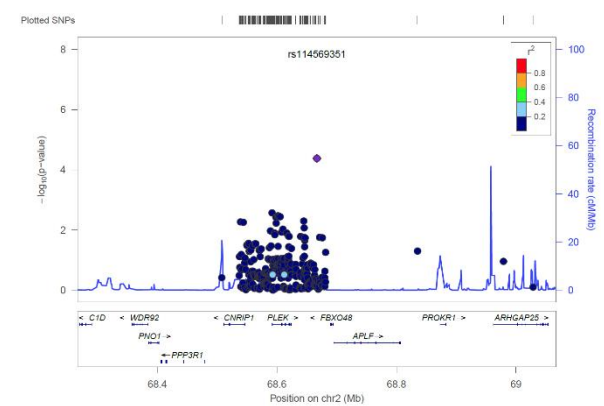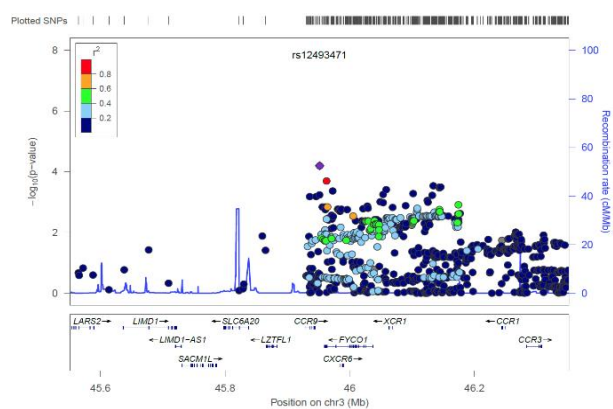

Supplement: S2 Fig — (PDF) [file pone.0152476.s008.pdf]
