# Supplementary material for: Identification of Non-HLA Genes Associated with Celiac Disease and Country-Specific Differences in a Large, International Pediatric Cohort
Source: PLoS One. 2016 Mar 25;11(3):e0152476. doi: 10.1371/journal.pone.0152476 (PMC4807782; doi:10.1371/journal.pone.0152476)
Supplement: S1 Text — (PDF) [file pone.0152476.s009.pdf]

Supplemental File

Section 2: TEDDY Study Acknowledgements;

All TEDDY sites and investigators

TEDDY Study Acknowledgements

The TEDDY Study Group (See appendix)

Funded by U01 DK63829, U01 DK63861, U01 DK63821, U01 DK63865, U01 DK63863, U01 DK63836, U01 DK63790, UC4 DK63829, UC4 DK63861, UC4 DK63821, UC4 DK63865, UC4 DK63863, UC4 DK63836, UC4 DK95300, and UC4 DK100238, and Contract No. HHSN267200700014C from the National Institute of Diabetes and Digestive and Kidney Diseases (NIDDK), National Institute of Allergy and Infectious Diseases (NIAID), National Institute of Child Health and Human Development (NICHD), National Institute of Environmental Health Sciences (NIEHS), Juvenile Diabetes Research Foundation (JDRF), and Centers for Disease Control and Prevention (CDC). This work supported in part by the NIH/NCATS Clinical and Translational Science Awards to the University of Florida (UL1 TR000064) and the University of Colorado (UL1 TR001082).

Appendix

The Teddy Study Group

Colorado Clinical Center: Marian Rewers, M.D., Ph.D., PI[1,4,5,6,10,11], Kimberly Bautista[12], Judith Baxter[9,10,12,15], Ruth Bedoy[2], Daniel Felipe-Morales, Brigitte Frohnert, M.D., Patricia Gesualdo[2,6,12,14,15], Michelle Hoffman[12,13,14], Rachel Karban[12], Edwin Liu, M.D.[13], Jill Norris, Ph.D.[2,3,12], Adela Samper-Imaz, Andrea Steck, M.D.[3,14], Kathleen Waugh[6,7,12,15], Hali Wright[12]. University of Colorado, Anschutz Medical Campus, Barbara Davis Center for Childhood Diabetes.

Georgia/Florida Clinical Center: Jin-Xiong She, Ph.D., PI[1,3,4,11,†], Desmond Schatz, M.D.*[4,5,7,8], Diane Hopkins[12], Leigh Steed[12,13,14,15], Jamie Thomas*[6,12], Katherine Silvis[2], Michael Haller, M.D.*[14], Meena Shankar*[2], Eleni Sheehan*, Melissa Gardiner, Richard McIndoe, Ph.D., Haitao Liu, M.D.†, John Nechtman†, Ashok Sharma, Joshua Williams, Gabriela Foghis, Stephen W. Anderson, M.D.^. Medical College of Georgia, Georgia Regents University. *University of Florida, †Jinfiniti Biosciences LLC, Augusta, GA, ^Pediatric Endocrine Associates, Atlanta, GA.

Germany Clinical Center: Anette G. Ziegler, M.D., PI[1,3,4,11], Andreas Beyerlein Ph.D.[2], Ezio Bonifacio Ph.D.*[5], Michael Hummel, M.D.[13], Sandra Hummel, Ph.D.[2], Kristina Foterek[¥2], Mathilde Kersting, Ph.D.[¥2], Annette Knopff[7], Sibylle Koletzko, M.D.[¶13], Claudia Peplow[12], Roswith Roth, Ph.D.[9], Joanna Stock[9,12], Elisabeth Strauss[12], Katharina Warncke, M.D.[14], Christiane Winkler, Ph.D.[2,12,15]. Forschergruppe Diabetes e.V. and Institute of Diabetes Research, Helmholtz Zentrum München, and Klinikum rechts der Isar, Technische Universität München. *Center for Regenerative Therapies, TU Dresden, ¶Dr. von Hauner Children´s Hospital, Department of Gastroenterology, Ludwig Maximillians University Munich, ¥Research Institute for Child Nutrition, Dortmund.

Finland Clinical Center: Jorma Toppari, M.D., Ph.D., PI[¥^1,4,11,14], Olli G. Simell, M.D., Ph.D., PI[¥^1,4,11,13], Annika Adamsson, Ph.D.[^12], Heikki Hyöty, M.D., Ph.D.[*±6], Jorma Ilonen, M.D., Ph.D.[¥ ¶3], Miia Kähönen[µ¤], Mikael Knip, M.D., Ph.D.[*±5], Annika Koivu[¥^], Mirva Koreasalo[*±§2], Kalle Kurppa, M.D., Ph.D.[*±13], Maria Lönnrot, M.D., Ph.D.[*±6], Elina Mäntymäki[¥^], Katja Multasuo[µ¤], Juha Mykkänen, Ph.D.[^¥ 3], Tiina Niininen[±*12], Mia Nyblom[*±], Petra Rajala[^], Jenna Rautanen[±§], Anne Riikonen[*±], Minna Romo[¥^], Satu Simell, M.D., Ph.D.[^±13], Tuula Simell, Ph.D., Ville Simell[^¥13], Maija Sjöberg[¥^12,14], Aino Stenius[µ¤12], Eeva Varjonen[¥^12], Riitta Veijola, M.D., Ph.D.[µ¤14], Suvi M. Virtanen, M.D., Ph.D.[*±§2], Mari Åkerlund[*±§]. [¥]University of Turku, *University of Tampere, [µ]University of Oulu, [^]Turku University Hospital, Hospital District of Southwest Finland, [±]Tampere University Hospital, [¤]Oulu University Hospital, §National Institute for Health and Welfare, Finland, [¶]University of Kuopio.

Sweden Clinical Center: Åke Lernmark, Ph.D., PI[1,3,4,5,6,8,10,11,15], Daniel Agardh, M.D., Ph.D.[13], Carin Andrén Aronsson[2,13], Maria Ask, Jenny Bremer, Ulla-Marie Carlsson, Corrado Cilio, Ph.D., M.D.[5], Camilla Ekstrand, Emelie Ericson-Hallström[2], Lina Fransson, Thomas Gard, Joanna Gerardsson, Rasmus Håkansson, Monica Hansen, Gertie Hansson[12], Susanne Hyberg, Fredrik Johansen, Berglind Jonasdottir M.D., Linda Jonsson, Helena Elding Larsson M.D., Ph.D. [6,14], Barbro Lernmark, Ph.D., Maria Månsson-Martinez, Maria Markan, Theodosia Massadakis, Jessica Melin[12], Zeliha Mestan, Kobra Rahmati, Anita Ramelius, Falastin Salami, Monica Sedig Järvirova, Sara Sibthorpe, Birgitta Sjöberg, Ulrica Swartling, Ph.D.[9,12], Erika Trulsson, Carina Törn, Ph.D. [3,15], Anne Wallin, Åsa Wimar[12,14], Sofie Åberg. Lund University.

Washington Clinical Center: William A. Hagopian, M.D., Ph.D., PI[1,3,4, 5, 6,7,11,13, 14], Xiang Yan, M.D., Michael Killian[6,7,12,13], Claire Cowen Crouch[12,14,15], Jennifer Skidmore[2], Stephen Ayres, Kayleen Dunson, Diana Heaney, Rachel Hervey, Corbin Johnson, Rachel Lyons, Arlene Meyer, Denise Mulenga, Emma Schulte, Elizabeth Scott, Joshua Stabbert, John Willis. Pacific Northwest Diabetes Research Institute.

Pennsylvania Satellite Center: Dorothy Becker, M.D., Margaret Franciscus, MaryEllen Dalmagro-Elias Smith[2], Ashi Daftary, M.D., Mary Beth Klein, Chrystal Yates. Children's Hospital of Pittsburgh of UPMC.

Data Coordinating Center: Jeffrey P. Krischer, Ph.D.,PI[1,4,5,10,11], Michael Abbondondolo, Sarah Austin-Gonzalez, Rasheedah Brown[12,15], Brant Burkhardt, Ph.D.[5,6], Martha Butterworth[2], David Cuthbertson, Christopher Eberhard, Steven Fiske[9], Dena Garcia, Veena Gowda, David Hadley, Ph.D.[3,13], Hye-Seung Lee, Ph.D.[1,2,13,15], Shu Liu, Xiang Liu, Ph.D.[2,9,12], Kristian Lynch, Ph.D. [5,6,9,15], Jamie Malloy, Cristina McCarthy[12,15], Wendy McLeod[2,5,6,13,15], Chris Shaffer, Laura Smith, Ph.D.[9,12], Susan Smith[12,15], Roy Tamura, Ph.D.[1,2,13], Ulla Uusitalo, Ph.D.[2,15], Kendra Vehik, Ph.D.[4,5,6,14,15], Ponni Vijayakandipan, Keith Wood, Jimin Yang, Ph.D., R.D.[2,15]. University of South Florida.

Project scientist: Beena Akolkar, Ph.D.[1,3,4,5, 6,7,10,11]. National Institutes of Diabetes and Digestive and Kidney Diseases.

Other contributors: Kasia Bourcier, Ph.D.[5], National Institutes of Allergy and Infectious Diseases. Thomas Briese, Ph.D.[6,15], Columbia University. Suzanne Bennett Johnson, Ph.D.[9,12], Florida State University. Steve Oberste, Ph.D.[6], Centers for Disease Control and Prevention. Eric Triplett, Ph.D.[6], University of Florida.

Autoantibody Reference Laboratories: Liping Yu, M.D.^[5], Dongmei Miao, M.D.^, Polly Bingley, M.D., FRCP*[5], Alistair Williams*, Kyla Chandler*, Saba Rokni*, Joanna Boldison*, Jacob Butterly*, Gabriella Carreno*, Claire Caygill*, Ivey Geoghan*, Anna Long*, Molly Payne*, James Pearson*, Sophie Ridewood*, Rebecca Wyatt*. ^Barbara Davis Center for Childhood Diabetes, University of Colorado Denver, *School of Clinical Sciences, University of Bristol UK.

Cortisol Laboratory: Elisabeth Aardal Eriksson, M.D., Ph.D., Ing-Marie Lundgren, Ewa Lönn Karlsson, Dzeneta Nezirevic Dernroth, Ph.D. Department of Clinical Chemistry, Linköping University Hospital, Linköping, Sweden.

Dietary Biomarkers Laboratory: Iris Erlund, Ph.D.[2], Irma Salminen, Jouko Sundvall, Jaana Leiviskä, Mari Lehtonen, Ph.D. National Institute for Health and Welfare, Helsinki, Finland.

HbA1c Laboratory: Randie R. Little, Ph.D., Alethea L. Tennill. Diabetes Diagnostic Laboratory, Dept. of Pathology, University of Missouri School of Medicine.

HLA Reference Laboratory: Henry Erlich, Ph.D.[3], Steven J. Mack, Ph.D., Anna Lisa Fear. Center for Genetics, Children's Hospital Oakland Research Institute.

Metabolomics Laboratory: Oliver Fiehn, Ph.D., Bill Wikoff, Ph.D., Brian Defelice, Dmitry Grapov, Ph.D., Tobias Kind, Ph.D., Mine Palazoglu, Luis Valdiviez, Benjamin Wancewicz, Gert Wohlgemuth, Joyce Wong. UC Davis Metabolomics Center.

Microbiome and Viral Metagenomics Laboratory: Joseph F. Petrosino, Ph.D.[6]. Alkek Center for Metagenomics and Microbiome Research, Department of Molecular Virology and Microbiology, Baylor College of Medicine.

OGTT Laboratory: Santica M. Marcovina, Ph.D., Sc.D., Vinod P. Gaur, Ph.D., Northwest Lipid Metabolism and Diabetes Research Laboratories, University of Washington.

Proteomics Laboratory: Richard D. Smith, Ph.D., Thomas O. Metz, Ph.D., Charles Ansong, Ph.D., Bobbie-Jo Webb-Robertson, Ph.D., and Hugh D. Mitchell, Ph.D. Pacific Northwest National Laboratory.

Repository: Heather Higgins, Sandra Ke. NIDDK Biosample Repository at Fisher BioServices.

RNA Laboratory and Gene Expression Laboratory: Jin-Xiong She, Ph.D., PI[1,3,4,11], Richard McIndoe, Ph.D., Haitao Liu, M.D., John Nechtman, Yansheng Zhao, Na Jiang, M.D. Jinfiniti Biosciences, LLC.

SNP Laboratory: Stephen S. Rich, Ph.D.[3], Wei-Min Chen, Ph.D.[3], Suna Onengut-Gumuscu, Ph.D.[3], Emily Farber, Rebecca Roche Pickin, Ph.D., Jordan Davis, Dan Gallo, Jessica Bonnie, Paul Campolieto. Center for Public Health Genomics, University of Virginia.

Committees:
[1]Ancillary Studies, [2]Diet, [3]Genetics, [4]Human Subjects/Publicity/Publications, [5]Immune Markers, [6]Infectious Agents, [7]Laboratory Implementation, [8]Maternal Studies,

[9]Psychosocial, [10]Quality Assurance, [11]Steering, [12]Study Coordinators, [13]Celiac Disease, [14]Clinical Implementation, [15]Quality Assurance Subcommittee on Data Quality.
